# Diagnosis and Management of Rare Case of Mesenteric Hematoma Rupture after Transcatheter Aortic Valve Replacement (TAVR): A Case Report and Review of the Literature

**DOI:** 10.1155/2018/6273538

**Published:** 2018-11-21

**Authors:** Danish Abbasi, Jeffrey E. Vanhook, Khashayar Salartash, Howard Levite

**Affiliations:** ^1^AtlantiCare Regional Medical Center, USA; ^2^Lewis Katz School of Medicine at Temple University, Department of Medicine, USA; ^3^American Heart Association for Central and Southern, New Jersey, USA

## Abstract

We present a case of a 78-year-old female with history of diastolic heart failure and paroxysmal atrial fibrillation on apixaban presenting with worsening shortness of breath. She underwent transesophageal echocardiogram showing severe aortic stenosis with a valve area of 0.8 cm2. Coronary angiography did not reveal significant coronary artery disease. CT of chest, abdomen, and pelvis did not show any evidence of hematoma or dissection. Patient was scheduled for transfemoral TAVR. Patient's apixaban was discontinued prior to the procedure. She received heparin during the procedure. She successfully underwent left transfemoral aortic valve replacement. Shortly after the procedure, she complained of abdominal pain and became hypotensive. Blood pressure was 76/44 mm of Hg (MAP 58). Hemoglobin dropped to 8.1 g/dl (baseline 13). Stat CT abdomen and pelvis showed a large volume of hemorrhage in the peritoneal cavity. CTA of abdomen showed no evidence of aortic aneurysm or dissection but active extravasation below the inferior aspect of the spleen. Catheterization of the superior mesenteric artery (SMA) identified ileal branch of SMA as the source of bleeding. Embolization using gel foam slurry followed by a coil insertion was performed. Repeat angiogram demonstrated continued extravasation through arcade collaterals. A rapid exploration of the abdominal cavity revealed ruptured mesenteric hematoma. Evacuation of hematoma was performed. Portion of small ileum and bleeding mesenteric branch vessel was resected. Her condition stabilized with no postoperative bleeding and she was discharged on warfarin postoperatively. Use of antithrombotic therapy increases risk of bleeding in TAVR patients. Mesenteric hematoma rupture if not identified can be life-threatening. We believe that this is the first reported case of mesenteric hematoma rupture after a TAVR procedure.

## 1. Introduction

TAVR/TAVI is increasingly used intervention in aortic stenosis patients. Bleeding is a common complication in TAVR patients. Mesenteric hematoma rupture is a rare clinical finding. We present a case of significant bleeding from a ruptured mesenteric hematoma after undergoing TAVR.

## 2. Case Presentation

A 78-year-old female with past medical history of chronic diastolic heart failure, pulmonary hypertension, paroxysmal atrial fibrillation on Eliquis (apixaban), left bundle branch block, coronary artery disease s/p DES to circumflex in 2010, myocardial infarction, hypertension, and hyperlipidemia presented with worsening shortness of breath. She underwent transesophageal echocardiogram which revealed a left ventricular ejection fraction of 55-60%. There was severe aortic stenosis and mild aortic regurgitation. The aortic valve area by continuity equation was 0.8 cm2. She was scheduled for cardiac catheterization which showed aortic pressure (Ao) 211/86, left ventricle (LV) 216/14, right atrium (RA) 8, pulmonary artery (PA) 43/20, and pulmonary wedge (PW) 21. Fick cardiac output was 4.22 and Fick cardiac index was 2.19. The aortic valve area was 0.85 cm2 with a mean gradient of 26 mmHg. The Langston measurements revealed a 22 mm peak to peak gradient with a mean gradient of 26 mm. The patient was hypertensive and had a moderately reduced cardiac output. Her aortic valve area index measured was 0.47. Coronary angiography revealed a right dominant system. There was 55% stenosis of the proximal LAD; however, instant flow reserve (IFR) was 0.90 and Fractional flow reserve (FFR) was 0.90 which did not show any need for PCI. 3-Mensio readings were used to estimate the calcium burden. Readings from both Medtronic and Edwards indicated significant valve calcification.

No provocative stress testing was done. CT of chest, abdomen, and pelvis as part of TAVR protocol did not show any evidence of hematoma or dissection. Patient blood workup showed normal renal and hepatic function levels (HgbA1C 5.6, Cr 0.89, Na 143, K 4.7, Albumin 3.8, ALK 90, AST 19, and ALT 28). CBC showed WBC 6.4, RBC 13.1, HCT 39.8, and Platelets 251. Coagulation profile was normal with PT 11.0, INR 1.1, and PTT 28.0.

Patient was scheduled for transfemoral TAVR. Patient's apixaban was discontinued prior to the procedure as per protocol. Patient's last dose of apixaban was 3 days prior to her procedure. Patient's blood pressure on presentation was 112/61, pulse rate 66, respiratory rate 14, and temperature of 97.5. Early during the TAVR procedure, the anesthesia record indicated a spike in BP to >200 mm. She had readings of SBP > 200 prior to her procedure. Patient BP was controlled with 10 mg IV of Hydralazine. Procedure was continued. Two Perclose devices were deployed in the left femoral artery, the vessel was dilated, and aortic valve replacement was performed. Valve was deployed under rapid ventricular pacing. Postdeployment assessment with hemodynamics and transesophageal echo aortography showed no aortic insufficiency. Patient received heparin during the procedure as per protocol. The patient received 14,000 units of heparin at the outset of the procedure. Protamine 140 mg was administered at the end of the procedure. The ACT peaked at 290. It began at 111 and was 119 at the end of the procedure. Patient was extubated and transferred to CVU.

**Figure 1:**
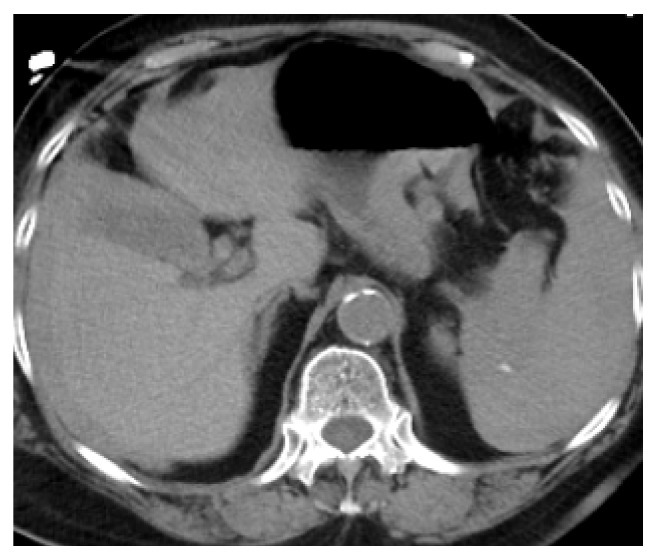
CT abdomen and pelvis without contrast showing hypodense area of bleeding.

**Figure 2:**
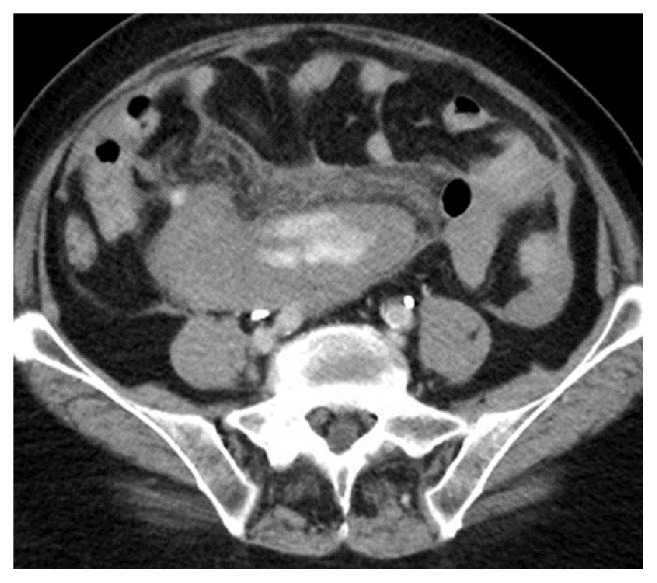
CTA abdomen showing hyperdense area of hemorrhage.

**Figure 3:**
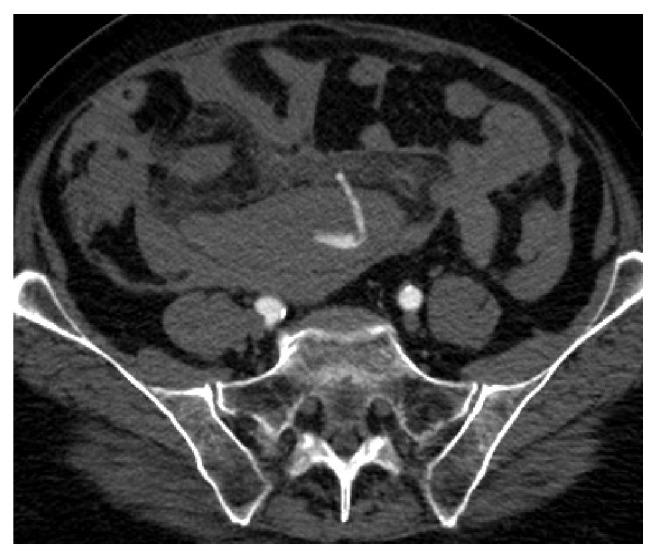
CTA abdomen hyperdense hemorrhaging vessel.

**Figure 4:**
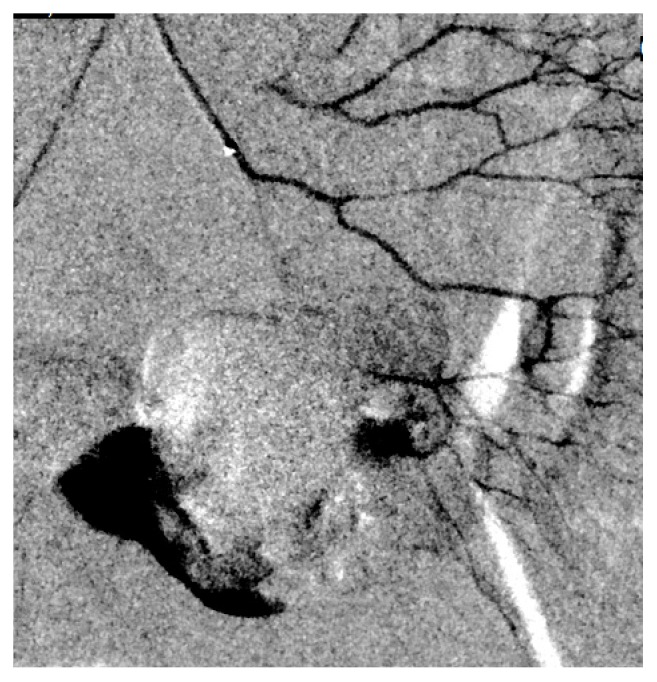
Angiogram showing extravasation from ileal branch of sma and early hematoma formation.

**Figure 5:**
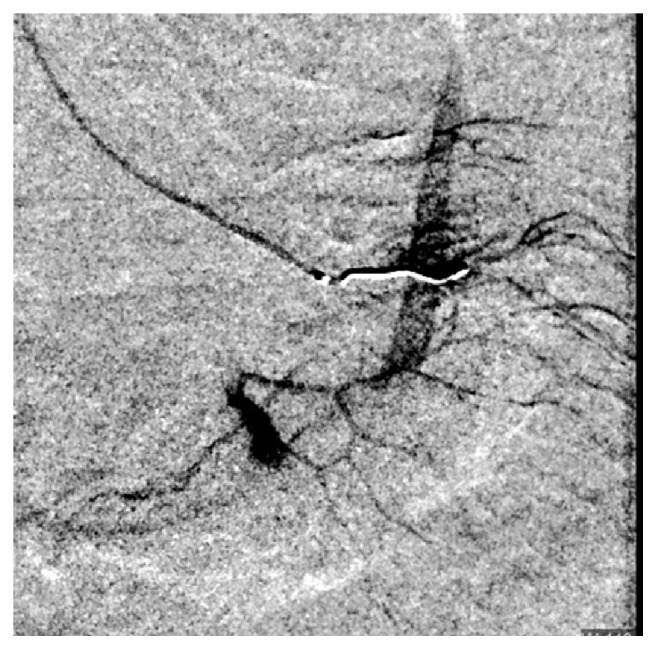
Angiogram showing coil insertion and continued extravasation from arcade branches.

Shortly after arriving in CVU patient complained of abdominal pain. She had abdominal discomfort on palpation. Patient subsequently became hypotensive. Her blood pressure dropped to 76/44 mm of Hg. Her MAP was 58mm of Hg. Patient's respiratory rate increased to 22 breaths per minutes. Echocardiogram showed hyperdynamic wall motion and volume depletion. Albumin was administered. She required increasing doses of neosynephrine. Patient's baseline hemoglobin was 13.1 g/dl with hematocrit of 39.8% which decreased to 8.1 g/dl with hematocrit of 23.7%. Blood transfusion was initiated. Patient underwent stat CT abdomen and pelvis which showed a large volume of hemorrhage in the peritoneal cavity (Figure 1). There was a focal hyper- dense area within the spleen. Computed tomography angiography (CTA) was performed which showed no evidence of aortic aneurysm or dissection. Superior mesenteric artery and inferior mesenteric artery were patent. There was active extravasation below the inferior aspect of the spleen which appeared to be the etiology for the large volume of hemoperitoneum (Figures 2 and 3). The patient was transferred to Interventional Radiology suite. Catheterization of the SMA was performed and mesenteric arteriography carried out. This demonstrated markedly diminutive vessels with active extravasation in the left lower quadrant of the abdomen from ileal branch. Ileal branch of superior mesenteric artery was identified as the source of bleeding (Figure 4). Selective catheterization of multiple ileal branches was performed using a microcatheter. This again demonstrated active extravasation from a vasa recta branch in the left lower quadrant. The microcatheter was advanced. Embolization was performed using gel foam slurry followed by a 2 mm x 1 cm coil. Repeat angiogram demonstrated thrombosis of the embolized branch but continued extravasation through arcade collateral (Figure 5).

Patient was transferred to OR. A rapid exploration of the abdominal cavity revealed distal ileal mesenteric hematoma of arterial branch. Evacuation of hematoma was performed. The mesentery itself was quite traumatized from the rupture of this mesenteric hematoma. The small intestine although viable was also affected significantly from the hematoma. Portion of small ileum was resected and reanastomosed. The bleeding mesenteric branch vessel was part of the resection. Patient was transitioned to critical care. Her condition started improving. She did not have any further bleeding complications. Patient was extubated successfully. Patient remained hemodynamically stable. She was slowly started on diet. Patient did continue to have loose watery stools postoperatively. Her C. difficile was negative. She was started on cholestyramine. She did not have any significant hemodynamic instability. Her hemoglobin remained stable. She was subsequently started on coumadin postoperatively. Patient was discharged to rehabilitation at postoperative day 9.

## 3. Discussion

Mesenteric Hematoma is a rare condition. Spontaneous hemoperitoneum as a term was first described by Barber in 1909 as a symptom associated with labor [1]. Due to its varied and nonspecific clinical presentation mesenteric hematomas are difficult to diagnose. No specific information is present regarding epidemiology. Most of the cases are associated with abdominal trauma. Information on nontraumatic mesenteric hematomas is limited to mainly case reports and retrospective literature reviews.

The potential causes of a nontraumatic mesenteric hematoma include anticoagulant therapy, connective tissue disease [2], acute pancreatitis [3] Crohn's disease [4], hemophilia [5], duodenal stenosis, and incarcerated inguinal hernia [6]. No reported cases of spontaneous mesenteric hematoma have been associated with uncontrolled HTN. Spontaneous mesenteric hematoma is classified as hematoma not associated with any cause [7, 8]. Hirano et al. considered nontraumatic hematoma associated with anticoagulation as spontaneous [9]. A review of cases in Japan concluded that in over half of the cases reported the cause was idiopathic.

According to Skudder et al. mesenteric hematomas suggest a rupture of a visceral artery aneurysm [10] and such findings warrant angiography. However, aneurysm of mesenteric arteries is uncommon [11]. In a review of 55 patients for vascular aneurysms, incidence of aneurysm of superior mesenteric artery was found to be 4% [12].

The symptoms are variable depending on clinical condition and may include abdominal pain, vomiting, hypotension due to bleeding. Abdominal mass can be palpated in cases of significant bleeding in to the abdomen. Weinstock et al. presented a case of small bowel obstruction resulting from extrinsic compression caused by mesenteric hematoma from spontaneous rupture of a jejunal branch artery [13]. These symptoms are not specific and need to be distinguished from related conditions that can cause intraperitoneal bleeding. Isolated aneurysms are usually asymptomatic and are generally undetected until they rupture causing significant bleeding [14]. Cases of mesenteric hematomas have been diagnosed in autopsies of patient dying from hemorrhagic shock [15].

The most used diagnostic methods are abdominal ultrasound and CT scan. Abdominal sonography in addition to color Doppler can be used to diagnose mesenteric hematoma [16]. CT scan is mostly commonly utilized imaging modality. CT reports must be interpreted with caution. CT appearance of hematoma also depends on how soon after the bleeding episode the patient is imaged. A fresh hematoma is seen as a dense lesion, while an old clotted hematoma will show up as a soft-tissue mass. [17]. A review of 17 cases of mesenteric hematoma in Japan by Aoki et al. correlated the CT findings of mesenteric hematoma with timing of diagnosis. In the first 2 weeks after bleeding most hematomas show increased density because of the high protein content of the entrapped hemoglobin. With progressive breakdown and removal of protein the attenuation value of hematoma reduces. The attenuation value of hematoma may reach that of serum in 2 to 4 weeks [18]. Mesenteric hematomas can sometimes be confused with tumors and are diagnosed on laparoscopic evaluation [19]. Gomez et al. reported two cases of spontaneous mesenteric hematoma diagnosed by MRI [20]. Angiography is used to localize the source of bleeding.

Management of patients with mesenteric hematoma depends on their clinical stability. Management can vary between conservative monitoring with interval imaging [20], embolization, or surgical intervention. Patients in shock not responding to fluid resuscitation need emergent surgery. Patients stable after resuscitation require urgent imaging. Corzo et al. reviewed 33 cases of mesenteric hematoma to identify clinical and radiological characteristics for operative management. The study recommended nonoperative management in patient with no abdominal pain, tenderness or free fluid [21]. Transcatheter Arterial Embolization is used as an alternative to surgery to control bleeding [22, 23]. Shin et al. published a study recommending transcatheter arterial embolization as a safe and effective minimally invasive alternative to surgery [24]

Severe aortic stenosis (AS) is a major cause of morbidity and mortality in the elderly. Prevalence of valvular heart disease increases with age. Metanalysis by Osnabrugge et al. [25] revealed a prevalence of 12.4% in the elderly (age ≥75 years). The prevalence of severe AS was 3.4%. Among elderly patients with severe AS, 75.6% were symptomatic, and 40.5% of these patients were not treated surgically.GI bleeds in patients with severe AS are usually associated with angiodysplasia. Association between aortic stenosis (AS) and gastrointestinal (GI) bleeding attributed to intestinal angiodysplasia has been termed Heyde's syndrome [26]. No data exists regarding presence of mesenteric hematomas in aortic stenosis.

TAVR (transcatheter aortic valve replacement) has emerged as an alternative option to surgical aortic valve replacement (SAVR) to treat severe, symptomatic aortic stenosis patients. Both transfemoral and transapical approaches were associated with comparable mortality for the treatment of patients at very high or prohibitive surgical risk [27]. A 2-year follow-up of patients in the PARTNER trial by Kodali et al. supported TAVR as an alternative to surgery in high-risk patients. TAVR was similar with respect to mortality, reduction in symptoms, and improved valve hemodynamics to surgical valve replacement [28].

Review of 663 patient with severe aortic stenosis after TAVI (transaortic valve implantation) by Tamburino et al. [29] showed cumulative incidences of mortality were 5.4% at 30 days, 12.2% at 6 months, and 15.0% at 1 year. Clinical and hemodynamic benefits observed acutely after TAVI were sustained at 1 year. Cardiac tamponade, conversion to open surgery, major access site complications, EF <40%, prior balloon valvuloplasty, and diabetes mellitus were independent predictors of mortality at 30 days.

Bleeding is a known and frequent complication of TAVR. A Weighted Meta-Analysis of 3,519 Patients from 16 Studies by Genereux et al. showed incidence of life-threatening bleeding at 15.6% [30]. RBC transfusion was associated with increased mortality at 1 year and increased risk of major stroke and acute kidney injury [31]. PARTNER Trial compared bleeding complications after surgical aortic valve replacement (SAVR) with transcatheter aortic valve replacement (TAVR). Major bleeding complication was identified as the strongest independent predictor of 1-year mortality. Bleeding complications were more common after SAVR than after TAVR and were also associated with a worse long-term prognosis [30]. Stanger et al. in a single center retrospective evaluation showed that TAVR is associated with a moderate risk of severe UGIB. Patients on triple antithrombotic therapy are at highest risk for severe UGIB [32]

Adjuvant antithrombotic therapies are used to decrease the risk of valve thrombosis and thromboembolic cerebrovascular during and after TAVR procedure. Type of anticoagulant used during the perioperative phase is variable. The BRAVO-3 Trial compared bivalirudin with heparin anticoagulation in transcatheter aortic valve replacement. Bivalirudin did not reduce rates of major bleeding at 48 h or net adverse cardiovascular events within 30 days compared with heparin [33]. Bernelli et al. reviewed the impact of baseline activated clotting time- (ACT-) guided heparin administration on major bleeding after transfemoral transcatheter aortic valve implantation [34]. Results showed that ACT-guided group had a significantly lower occurrence of major, life-threatening, and any bleeding. Based on these findings 2012 joint consensus document favored an activated clotting time (ACT) of >300 seconds [35].

A meta-analysis by Aryal et al. showed no difference in risk of stroke or MI at 30 days following TAVR with use of single antiplatelet therapy versus dual antiplatelet therapy (DAPT). DAPT use may be associated with a higher bleeding risk [36]. The ARTE (Aspirin Versus Aspirin + Clopidogrel Following Transcatheter Aortic Valve Implantation) Randomized Clinical Trial also showed similar findings [37]. In atrial fibrillation patients use of coumadin with concomitant antiplatelet therapy did not appear to reduce the incidence of stroke, major adverse cardiovascular events, or death, while increasing the risk of major or life-threatening bleeding [38].

Use of oral anticoagulation in TAVR patient with atrial fibrillation remains variable. No consensus guidelines exist. Based on the RE-ALIGN trial data [39] new oral anticoagulants are not recommended in patients with valvular heart disease; however definition on Valvular AF remains unclear. Oral vitamin K inhibitor along with warfarin remains the most commonly used regimen [40]. Multiple trials are currently ongoing to assess the safety of oral anticoagulation in TAVR patients. The results so far have been variable. DAWA pilot study evaluated the use of dabigatran in patients with bioprosthetic valve replacement and AF. Use of dabigatran appeared similar to warfarin in preventing the formation of intracardiac thrombus; however trial was terminated prematurely because of low enrollment [41]. In Galileo trial patients were randomized to either a rivaroxaban-based strategy or an antiplatelet-based strategy [42]. The trial's data safety monitoring board recommended discontinuing the trial after the study showed that patients treated with rivaroxaban had higher rates of outcome of death or thromboembolic event (11.4% versus 8.8%), as well as the primary safety outcome of primary bleeding (4.2% versus 2.4%). All-cause death rates were also higher with rivaroxaban (6.8% versus 3.3%). The FRANCE-TAVI registry evaluated the role of oral anticoagulation in long-term survival and early bioprosthetic valve dysfunction (BVD) in patients who underwent successful TAVI. Use of oral anticoagulation was associated with decreased BVD but was associated with mortality independently of atrial fibrillation [43]. In absence of definitive data further research is required for definitive recommendations regarding anticoagulation in AF patients with TAVR.

## 4. Conclusion

Bleeding is a known complication of TAVR procedure associated with higher mortality. Use of adjuvant antithrombotic therapy increases risk of bleeding. Mesenteric hematoma rupture is a very rare and potentially life-threatening complication. We present to the best of our knowledge the first reported case of mesenteric hematoma rupture after a TAVR procedure.

## Data Availability

All the procedures (TAVR, Angiogram, and exploratory laparotomy) mentioned in the case report are approved for the case as per guidelines. Relevant citations have been provided in the article. Any specific details can be provided upon request from AtlantiCare Regional Medical Center and corresponding author.
